# A Novel 2-Carbon-Linked Dimeric Artemisinin With Potent Antileukemic Activity and Favorable Pharmacology

**DOI:** 10.3389/fonc.2021.790037

**Published:** 2022-01-11

**Authors:** Amanda B. Kagan, Blake S. Moses, Bryan T. Mott, Ganesha Rai, Nicole M. Anders, Michelle A. Rudek, Curt I. Civin

**Affiliations:** ^1^ Department of Medicine, Division of Clinical Pharmacology, School of Medicine, Johns Hopkins University, Baltimore, MD, United States; ^2^ Center for Stem Cell Biology & Regenerative Medicine, Marlene and Stewart Greenebaum Comprehensive Cancer Center, Departments of Pediatrics and Physiology, School of Medicine, University of Maryland, Baltimore, MD, United States; ^3^ Department of Neurosurgery, Wake Forest Baptist Health, Winston-Salem, NC, United States; ^4^ Department of Pre-Clinical Innovation, National Center for Advancing Translational Sciences, National Institutes of Health, Bethesda, MD, United States; ^5^ Department of Oncology, School of Medicine, Johns Hopkins University, Baltimore, MD, United States

**Keywords:** artemisinins, sorafenib, venetoclax (ABT-199), leukemia, antineoplastics

## Abstract

Acute myeloid leukemia (AML) remains a devastating disease, with low cure rates despite intensive standard chemotherapy regimens. In the past decade, targeted antileukemic drugs have emerged from research efforts. Nevertheless, targeted therapies are often effective for only a subset of patients whose leukemias harbor a distinct mutational or gene expression profile and provide only transient antileukemic responses as monotherapies. We previously presented single agent and combination preclinical data for a novel 3-carbon-linked artemisinin-derived dimer (3C-ART), diphenylphosphate analog 838 (ART838), that indicates a promising approach to treat AML, given its demonstrated synergy with targeted antileukemic drugs and large therapeutic window. We now report new data from our initial evaluation of a structurally distinct class of 2-carbon-linked dimeric artemisinin-derived analogs (2C-ARTs) with prior documented *in vivo* antimalarial activity. These 2C-ARTs have antileukemic activity at low (nM) concentrations, have similar cooperativity with other antineoplastic drugs and comparable physicochemical properties to ART838, and provide a viable path to clinical development.

## Introduction

The prognosis for patients with acute myeloid leukemia (AML) remains poor (5-year survival ~ 25-30%) and is particularly dismal for patients who are over 60 years old, unfit, or with relapsed/refractory disease, unfavorable cytogenetics, or certain molecular abnormalities (1). According to the Surveillance, Epidemiology, and End Results (SEER) database from 2010-2017, median overall survival in *de novo* AML was only 11 months (2). Improved understanding of the pathophysiology and mutational landscape of AML has stimulated the successful development of a number of targeted therapies. In fact, nine new AML therapeutics – including inhibitors of B-cell lymphoma 2 (BCL2), FMS-like tyrosine kinase 3 (FLT3), and isocitrate dehydrogenase (IDH) – were approved by the FDA between 2017 and 2021 (1, 3). However, the use of these targeted inhibitors as monotherapies has achieved only shallow, transient responses followed by the emergence of therapy resistant AML in the majority of patients with targetable mutations (4–6). There has thus been growing interest in additional new agents and combination regimens with novel mechanisms that may be non-cross resistant with respect to current antileukemic drugs, and that suggest low clinical toxicity.

**Figure 1:**
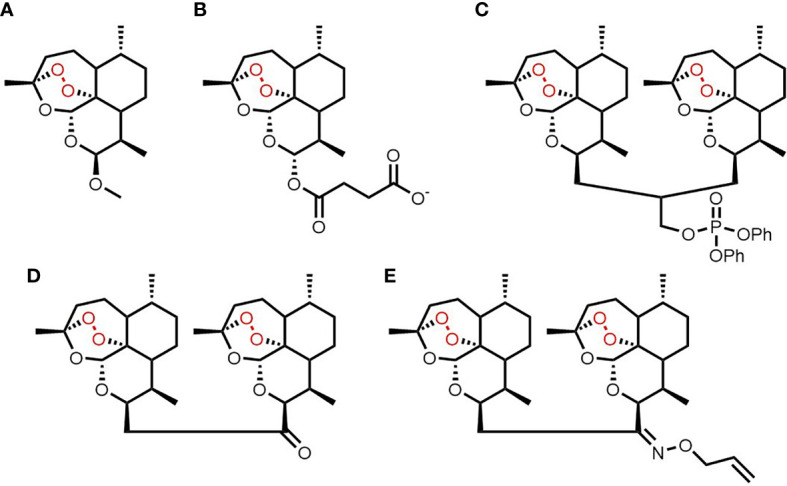
Chemical structures of artemisinin **(A)**, artesunate **(B)**, ART838 **(C)**, ART576 **(D)** and ART631 **(E)**.

The revolutionary antimalarial natural product artemisinin and its semi-synthetic derivatives (collectively referred to here as ARTs) (**Figure 1**) all share a unique endoperoxide pharmacophore that is bio-activated to cytotoxic carbon free radical species (7). Although they are best known as antimalarials, ARTs are active against a range of microbes (8–10) and also have potent antineoplastic activity (11–14). They are active against a broad array of leukemia cell lines, including those harboring poor risk genetic aberrations, such as FLT3 mutations and BCR-ABL and mixed-lineage leukemia (MLL) rearrangements, and those resistant to current antileukemic drugs (15–18). First-generation ART derivatives suffer from poor bioavailability and rapid metabolism (7), precluding the sustained exposure desired for antineoplastic therapy. Semi-synthetic trioxane dimers were designed to address these pharmacokinetic vulnerabilities (19). We previously reported that the novel 3-carbon linked ART dimer (3C-ART), diphenylphosphate analog 838 (ART838), was 88-fold more potent than artesunate (AS), the major clinical artemisinin derivative, against 23 leukemia cell lines, with a favorable therapeutic window (17). ART838 synergized with the kinase inhibitor sorafenib (SOR) and the BCL2 inhibitor venetoclax (VEN) (to form a combination regimen referred to as “SAV”) both *in vitro* and in AML xenograft and PDX models (20). Unfortunately, commercial development of ART838 is impractical, as its patent protection ends soon (US7417156B2 issued 9/27/02).

A structurally distinct class of two-carbon linked ART dimers (2C-ARTs) was originally developed to further enhance drug-like properties (19). We evaluated two of over 20 analogs from this 2C-ART class that were shown to be effective against an *in vivo* mouse model of malaria (19). Ketone analog ART576 and allyloxime analog ART631 were selected for evaluation due to their antimalarial efficacy and ease of synthesis. In addition, for both these ART analogs, there was unpublished *in vitro* data demonstrating potent activity against a range of cancer cell lines, including several leukemia cell lines. ART576 and ART631 (**Figure 1**) were evaluated for *in vitro* and *in vivo* antileukemic efficacy, both as monotherapies and in combination with SOR and VEN, as well as in pharmacodynamic and pharmacokinetic (PK) assays.

## Materials and Methods

### Reagents

Synthesis of 2C-ARTs has been described previously (19). All other compounds, antibodies, and reagents were as detailed in Supplemental Table 1 of Moses et al. (20).

### Cells

The AML cell lines used for *in vitro* antileukemic activity assays are described in **Table 1**. Lentiviral-transduced luciferase (luc)-labeled MV4;11 and MOLM14 AML cells were developed and characterized previously (20, 21)

**Table 1:** AML cell lines.

Cell Line	Major Genetic Aberrations	Source	Catalog #	Culture Media
K562	BCR-ABL, TP53	ATCC, Manassas, VA	CCL-243	RPMI+10%FBS
KG1a	FGFR1OP2-FGFR1, TP53	ATCC, Manassas, VA	CCL-240	RPMI+10%FBS
HEL	JAK2V617F	ATCC, Manassas, VA	TIB-180	RPMI+10%FBS
HL60	PML-RARA, c-MYC	ATCC, Manassas, VA	CCL-246	RPMI+10%FBS
ML2	KMT2A-MLLT4, KRAS, TP53	DSMZ, Branschweig, DEU	ACC-15	RPMI+10%FBS
MOLM14	KMT2A-MLLT3, FLT3ITD, CBL Δexon 8	DSMZ, Branschweig, DEU	ACC-777	RPMI+10%FBS
MV4;11	KMT2A-AFF1, FLT3ITD	ATCC, Manassas, VA	CRL-9591	RPMI+10%FBS
TF1	EpoR truncation, IDH2	ATCC, Manassas, VA	CCL-2003	RPMI+10% FBS+0.5uL/mL GMCSF
THP1	KMT2A-MLLT3, NRAS, TP53	ATCC, Manassas, VA	TIB-202	RPMI+10% FBS+50uM 2-Mercaptoethanol
U937	PICALM-AF10, TP53	ATCC, Manassas, VA	CRL-1593	RPMI+10%FBS

These AML cell lines utilized for in vitro antileukemic activity testing (IC_50_) were purchased from the listed sources and cultured in the indicated media. Their major genetic aberrations, including oncogenic gene fusions, FLT3ITD status, and point mutations, are as listed.

### Cytotoxicity

Cytotoxicity was assessed using alamarBlue assays (Life Technologies, Grand Island, NY) following manufacturer’s guidelines. Cell death was verified by Annexin V/7-aminoactinomycin D (7AAD) staining (BioLegend, San Diego, CA) per manufacturer’s guidelines.

### Metabolic Stability

The metabolic stability of ART631 was assessed in human and mouse plasma and liver microsomes (LM). For phase I microsomal stability assays, analogs (10 µM in <1% acetonitrile) were incubated in PBS containing liver microsomes, with or without cofactors (22). Microsomal controls (without cofactors) were used to determine non-enzymatic degradation. All experiments were incubated at 37°C for 1h and performed in duplicate. Reactions were stopped with acetonitrile and analyzed as described below.

### Reactive Oxygen Species (ROS)

Cells were pre-treated with 5 mM ROS detection reagent 5-(and 6-)-chloromethyl-2’,7’-dichlorodihydrofluorescein diacetate (CM-H2DCFDA, Life Technologies) for 30 min, followed by the iron chelator deferoxamine mesylate (DFO; 11 mM) for 60 min. Cells were then treated with ART631 (200 nM) or DMSO control for 18h, with subsequent analysis by flow cytometry. Western blotting and quantitative reverse transcription PCR (qRT-PCR) to assess for molecular markers of ROS-mediated apoptosis were performed as previously described (20).

### Pharmacokinetics (PK)

Mice were dosed orally with ART631 at empirically determined maximally tolerated doses (MTDs) of 75 mg/kg ART631 as a single dose alone or 15 mg/kg as a multiple dose regimen (once daily x5), either alone or in combination with SOR (30 mg/kg/d) and VEN (150 mg/kg/d), for side-by-side comparison to the ART838, SOR and VEN combination evaluated previously (20). ART631, SOR, and VEN were mixed in vehicle and given as a single administration. Mice (n = 3/time point) were euthanized at 0.5, 1, 1.5, 2, 3, 4, 6, 8 and 12 h after dosing. ART631 was quantified in plasma as described below. Pharmacokinetic parameters were calculated from mean concentration-time data using non-compartmental methods in Phoenix WinNonlin version 8.3 (Certara, Princeton, NJ). The maximum plasma concentration (C_max_) and time to C_max_ (T_max_) were the observed values. The AUC_last_ was calculated using the log-linear trapezoidal method. AUC was extrapolated to infinity (AUC_INF_) by dividing the last quantifiable concentration by the terminal disposition rate constant (λ_z_). The λ_z_ was determined from at least 3 points on the slope of the terminal phase of the concentration-time profile. The terminal half-life (T_1/2_) was determined by dividing 0.693 by λ_z_. Apparent clearance (Cl/F) was calculated by dividing the dose administered by AUC_INF_. Apparent volume of distribution (V/F) was calculated by dividing Cl/F by λ_z_. If the percent AUC extrapolated was >20% or the r^2^ of λ_z_ was <0.85, the AUC_INF_, Cl/F, T_1/2_ and V/F were not reported.

The Method of Bailer was used to estimate the variance of AUC given the calculated variance of the mean concentration at each time point (23). A pairwise comparison utilizing Z test was used to determine whether there was a significant difference between ART631 exposures as expressed by AUC_last_ (24). In all cases, p < 0.05 was considered statistically significant.

### Bioanalytical Method

ART631 was extracted from 25 µL plasma or microsomal solutions with 100 µL acetonitrile containing artemisinin (i.e. the natural product) as internal standard. Chromatographic separation was achieved with a Zorbax XDB C18 column (2.1 x 50mm, 3.5µm, Agilent Technologies, Santa Clara, CA) at room temperature using gradient elution over a 6 min total analytical run time. Mobile phase A was ammonium acetate (2mM) containing 0.1% formic acid, and mobile phase B was acetonitrile containing 0.1% formic acid. The gradient started with mobile phase B and was held at 40% for 0.5 minutes with a flow rate of 0.4 mL/min, then increased to 100% over 1.5 minutes and held for 2.0 minutes, and finally returned back to 40% mobile phase B and allowed to equilibrate for 2.0 minutes. An AB Sciex 5500 triple quadrupole mass spectrometer operated in positive electrospray ionization mode was used for the detection of ART631. For stability studies, the ratio of ART631 to the internal standard, artemisinin, was assessed. Calibration curves for ART631 were computed using the ratio of the peak area to that of the internal standard by using a quadratic equation with a 1/x^2^ weighting function over the range of 8-1585 nM with dilutions of up to 1:10 (v/v).

### AML (Cell Line) Xenograft and Patient-Derived Xenograft (PDX) Assays

Immunodeficient NOD-*Rag1^null^IL2rg^null^* (NRG) mice were purchased from Jackson Laboratory (Bar Harbor, ME), bred and housed at the University of Maryland Baltimore. Similar to our previous publication (20). mice were transplanted IV with luc-labeled human AML cell lines or cryopreserved AML patient cells on day -10, and baseline total body luminescence was quantified on day 0 by Xenogen bioluminescence imaging (Xenogen IVIS Spectrum; PerkinElmer, Waltham, MA). Mice were allocated to treatment groups so that each group had similar average day 0 luminescence, then groups were administered drugs per oral (PO; gavage). Luminescence of each mouse was assessed over time and compared with that mouse’s day 0 luminescence (fold-change AML burden). Clinical behavior, appearance, weight, and survival were also monitored.

## Results

### ART576 and ART631 Exhibited *In Vitro* Activity Against Multiple AML Cell Lines at Nanomolar Concentrations

**Figure 2:**
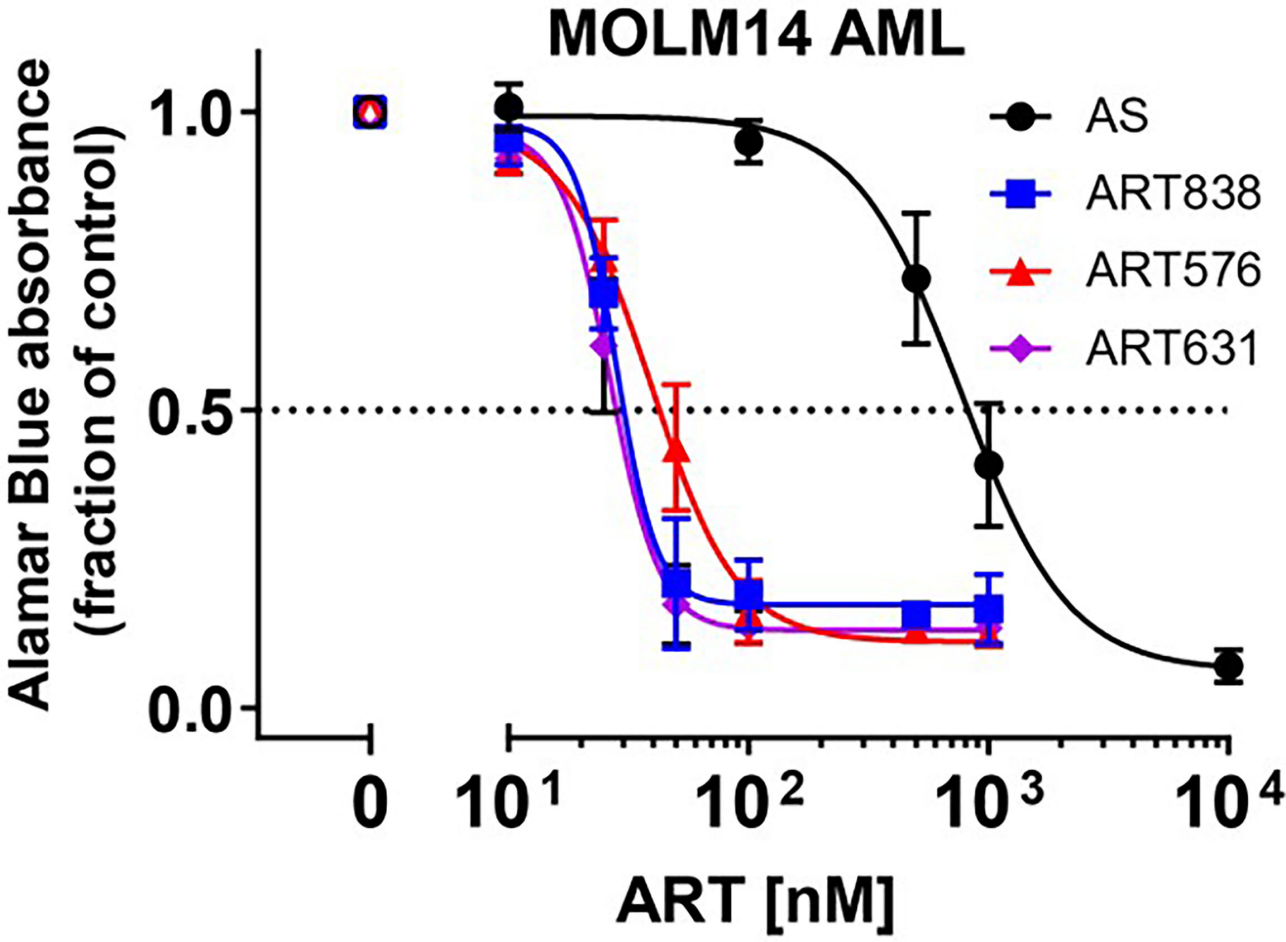
2C-ARTs ART631 and ART576 potently inhibited the growth of MOLM14 AML cells, with activity comparable to ART838. In this experiment, provided to show the entire concentration:response range, MOLM14 AML cells were cultured with a range of concentrations of artesunate (AS), ART838, ART631, and ART576 for 48h. Graph indicates growth inhibition relative to vehicle (DMSO)-treated controls by alamarBlue absorbance assays. Data points and error bars represent mean and SD of 3 independent experiments done in triplicate. Although **Table 1** shows only IC_50_s, this full concentration:response range was assessed for all 10 AML cell lines.

In alamarBlue assays, ART838, ART631, and ART576 had comparable *in vitro* activity against the human MOLM14 AML cell line, and all 3 were substantially more potent than the first-generation derivative artesunate (AS) (**Figure 2**). ART576 inhibited growth of 8 of 10 human AML cell lines tested, and ART576’s antileukemic potency (IC_50_ range: 23-105 nM; **Table 2**) was similar to that of ART838, which inhibited 9 of the same 10 cell lines (IC_50_ range: 22-112 nM). ART576 did not inhibit THP1 cell growth (IC_50_ >1000 nM), while ART838 did (IC_50_ = 45 nM). ART631 inhibited growth of the same 9 of these 10 human AML cell lines (IC_50_ range: 20-45 nM; **Table 2** **)** as did ART838, slightly exceeding the potency of ART838. Cell lines with adverse risk cytogenetic and molecular features, e.g. MLL rearrangements (ML2, MOLM14, MV4;11, THP1), FLT3-ITD (MOLM14, MV4;11), TP53 mutations (KG1a, ML2, THP1, U937), and NRAS/KRAS mutations (ML2, THP1), were among those exquisitely sensitive to ART631, with IC_50_s < 50 nM. The K562 cell line was the only exception, as it was resistant to all 3 of these ARTs. Based on this *in vitro* activity data combined with the drug stability data described below, ART631 was prioritized over ART576 for further *in vitro* and *in vivo* antileukemic efficacy experiments here (**Table 2**).

**Table 2:** *In vitro* IC_50_ values for ART analogs against 10 human AML cell lines.

AML	IC_50_ (nM)			
	ART631	ART576	ART838	AS
K562	>1000	>1000	>1000	>10000
KG1a	29±2	64±4	112±7	2603±458
HEL	45±5	80±19	72±6	1114±93
HL60	41±9	72±18	45±3	1057±83
ML2	34±4	105±12	40±5	1578±94
MOLM14	32±4	43±7	28±3	890±99
MV4;11	27±2	38±10	28±1	610±30
TF1	27±2	49±12	36±3	1715±302
THP1	42±2	>1000	45±3	>10000
U937	20±5	23±1	22±3	521±96

AML cell lines were cultured with a range of concentrations of AS, ART838, ART631, or ART576 for 48h. IC_50_s were calculated based on observed growth inhibition relative to vehicle (DMSO)-treated controls using alamarBlue absorbance assays (means of 2 independent alamarBlue assays performed with triplicate samples ± SD).

### ART631 Was More Stable Than ART576 in Microsomes

We compared the *in vitro* stability of ART576 and ART631 (**Table 3**). ART576 was stable in plasma (<10% degradation), but unstable with degradation of >70% in human liver microsomes with co-factors and >90% in mouse liver microsomes with co-factors (**Table 3**). Compared to ART576, ART631 was less stable in plasma with degradation of >40%, but was considerably less susceptible to NADPH-dependent phase I metabolism, with ~30% degradation in human liver microsomes with co-factors and ~40% in mouse liver microsomes with co-factors (**Table 3**). Based on these results as well as the *in vitro* antileukemic activity results above, we prioritized ART631 over ART576 for *in vivo* PK profiling and antileukemic efficacy assays, despite the fact that neither were equivalent to ART838 in stability [≤20% degradation as previously reported (20)].

**Table 3:** Stabilities of ARTs.

Matrix	Plasma (EDTA)		Phase I Metabolism			
Cofactors	Mouse	Human	MLM		HLM	
			NADPH(-)	NADPH(+)	NADPH(-)	NADPH(+)
**ART576**	101	91	96	7	89	26
**ART631**	62	60	60	35	86	60

2C-ART analogs were compared for stability in human and mouse plasma or in LM with or without cofactors. All values represent percentages remaining of the original ART analog.

### ART631 Synergized With the Kinase Inhibitor Sorafenib (SOR) and the BCL2 Inhibitor Venetoclax (VEN) to Cause Apoptotic AML Cell Death

**Figure 3:**
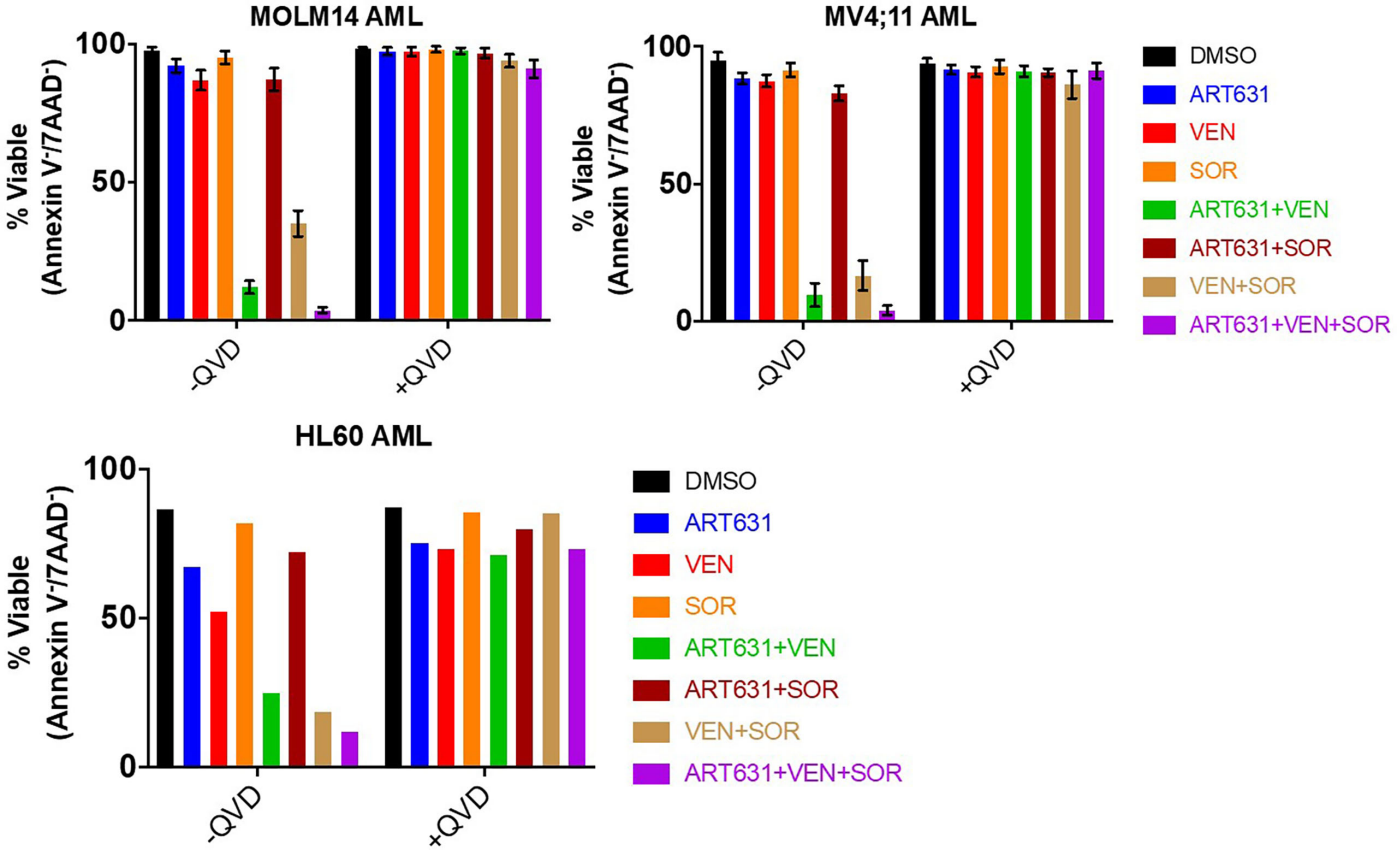
ART631-based combination treatments reduced cell viability in AML cell lines. MOLM14, MV4;11, and HL60 AML cells were each treated for 18h with vehicle (DMSO) control, ART631 alone (200 nM), VEN alone (50 nM), SOR alone (5 uM), and 2- and 3-drug combinations, all with and without a 30-45 min pre-incubation with 10 nM pan-caspase inhibitor QVD-OPh (QVD). Treated cells were stained with 2.5 ug/mL 7-aminoactinomycin D (7-AAD) and 0.5 ug/mL allophycocyanin (APC)-conjugated Annexin V in Annexin V binding buffer and analyzed by flow cytometry. Graphs indicate % cell viability based on Annexin V/7-AAD dual staining. For MOLM14 and MV4;11, means and SDs shown are based on 3 independent experiments. HL60 cell data is from one experiment done in triplicate.

MOLM14, MV4;11, and HL60 human AML cells were assessed for cell viability and caspase-dependent apoptosis following treatment with ART631 (200 nM), VEN (50 nM), SOR (5 µM), and combinations thereof. These concentrations were chosen because they resulted in less than 15% cell death in these cell lines when drugs were given as single agents (20). Consistent with published data for ART838 (20), there was substantially more synergy between ART631 and VEN than between ART631 and SOR in all three cell lines, and the VEN plus SOR combination was also highly effective (**Figure 3**). Importantly, treatment with ART631 plus VEN plus SOR as a 3-drug combination induced apoptotic cell death more potently than any of the 2-drug combinations, although there was only modest superiority of the ART631 plus VEN plus SOR 3-drug combination over the ART631 plus VEN or the VEN plus SOR 2-drug combinations (**Figure 3**). Pre-treatment with the pan-caspase inhibitor QVD-OPh (QVD) almost fully inhibited apoptosis and cell death for all of these drugs and combinations.

### ART631 Elevated ROS and DDIT3/CHOP Levels, and Reduced MCL1 Protein Levels in AML Cells

**Figure 4:**
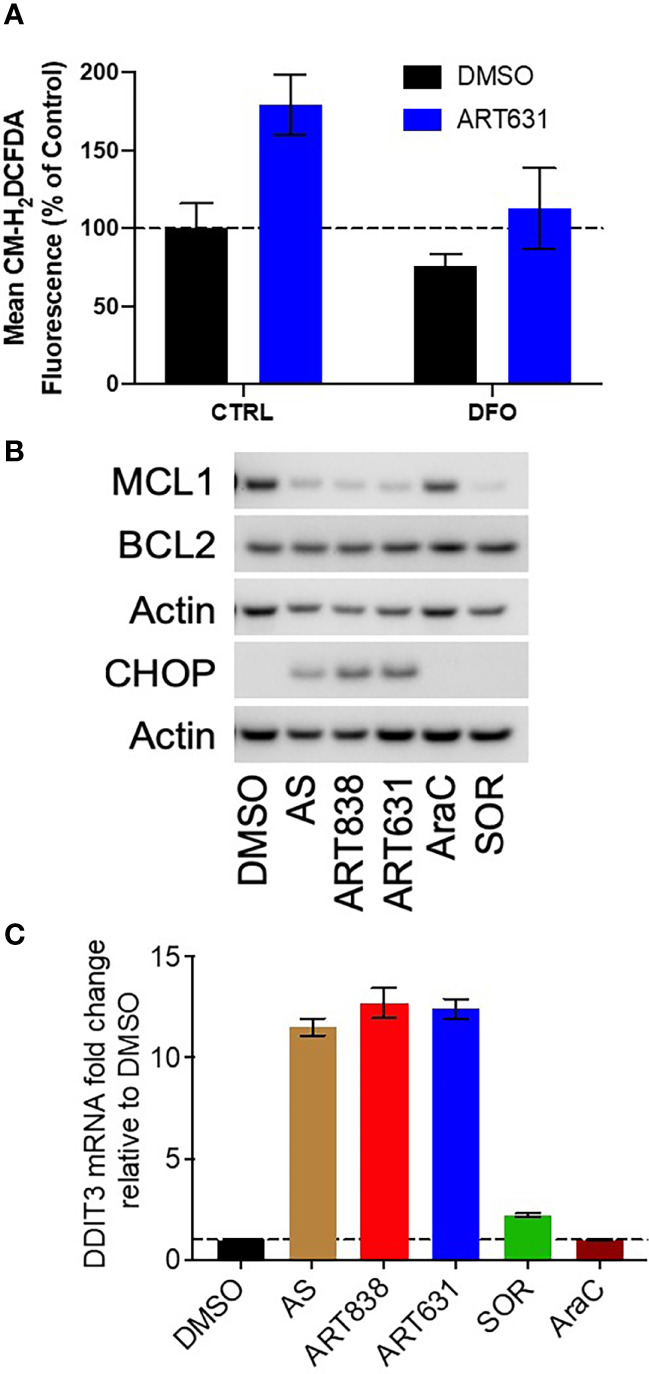
ART631 elevated ROS and CHOP levels and reduced MCL1 expression in MOLM14 AML cells. **(A)** MOLM14 AML cells were pre-treated with ROS detection reagent CM-H2DCFDA (5 mM) for 30 min, pre-treated with deferoxamine mesylate (DFO; 11 mM) for 60 min, and then treated with ART631 (200 nM) or DMSO control for 18h. They were then analyzed by flow cytometry. Graph represents %CM-H2DCFDA fluorescence compared to vehicle (DMSO) control treated cells. Error bars represent SD from 3 independent experiments. **(B)** MOLM14 AML cells were treated for 18h with vehicle, AS (10 uM), ART838 (200 nM), ART631 (200 nM), cytarabine (AraC; 150 nM), or SOR (5 uM). Protein was isolated and western blotted for MCL1, BCL2, CHOP, and β-actin. **(C)** MOLM14 cells were treated as in **(B)** for 18h, total RNA isolated, cDNA synthesized, and SYBR Green qRT-PCR performed in triplicates. Ct values were normalized to housekeeping gene GAPDH and fold change shown relative to vehicle (DMSO) control. Error bars represent SD from 3 independent experiments done in triplicate.

**Figure 5:**
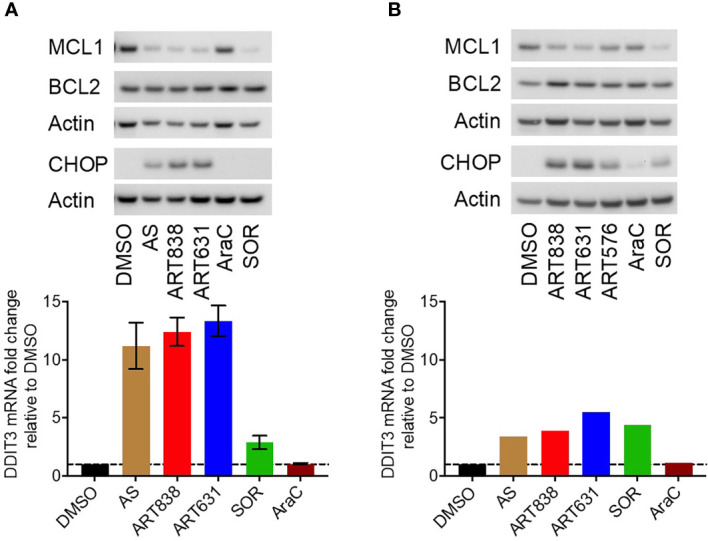
ART631 elevated ROS and CHOP levels and reduced MCL1 expression in MV4;11 and ML2 AML cells. **(A)** MV4;11 cells (left) and ML2 cells (right) were treated for 18h with vehicle, AS (10 uM), ART838 (200 nM), ART631 (200 nM), cytarabine (AraC; 150 nM), or SOR (5 uM). Protein was isolated and western blotted for MCL1, BCL2, CHOP, and β-actin. **(B)** MV4;11 cells (left) and ML2 cells (right) were treated as in **(A)** for 18h, total RNA isolated, cDNA synthesized, and SYBR Green qRT-PCR performed. Ct values were normalized to housekeeping gene GAPDH and fold change shown relative to vehicle (DMSO) control. Error bars represent SD from 3 independent experiments done in triplicate. ML2 cell data is from one experiment done in triplicate.

The accepted antimicrobial and antineoplastic mechanism of ARTs involves bioactivation of the endoperoxide pharmacophore by Fe^2+^-containing molecules, resulting in generation of reactive oxygen species (ROS) and free radicals (25). Consistent with this mechanism, treatment of MOLM14 cells with ART631 (200 nM) substantially increased total cellular ROS levels (**Figure 4A**), and pre-treatment with the iron chelator deferoxamine mesylate (DFO; 11 uM) diminished ROS generation by ART631, as demonstrated previously for ART838 (17). There was an associated increase in the level of C/EBP homologous protein (CHOP), a protein well known to play an important role in ROS-induced apoptosis (18), following treatment with ART631 (**Figure 4B**), comparable to that observed with ART838. Consistent with CHOP protein elevation, DDIT3, the mRNA which encodes CHOP, was increased in MOLM14 cells treated with ART631, ART838, or AS, by qRT-PCR (**Figure 4C**), suggesting DDIT3 as an attractive potential pharmacodynamic biomarker. Last, like ART838 and other ARTs (18, 20), ART631 decreased anti-apoptotic protein MCL1 expression substantially, by western blotting of MOLM14 cells (**Figure 4B**). Western blotting to assess CHOP and MCL1 expression and qRT-PCR to assess DDIT3 mRNA levels were also performed in MV4;11 and ML2 AML cells with similar results (**Figures 5A, B** **)**.

### Maximum Tolerated Dose (MTD) and PK of ART631 in NRG Mice

In pilot experiments, the MTD of ART631 was determined, in NRG (host strain for our AML xenograft models) mice, to be 75 mg/kg PO (gavage) for a single dose, or 15 mg/kg/d PO x5d every 14 days. When ART631 was combined with 150 mg/kg/d VEN and/or 30 mg/kg/d SOR, as was done previously with ART838 (20), ART631 was tolerated at the multiple dose MTD, and no further dose adjustments were made. The PK of ART631 was assessed at the MTD.

**Figure 6:**
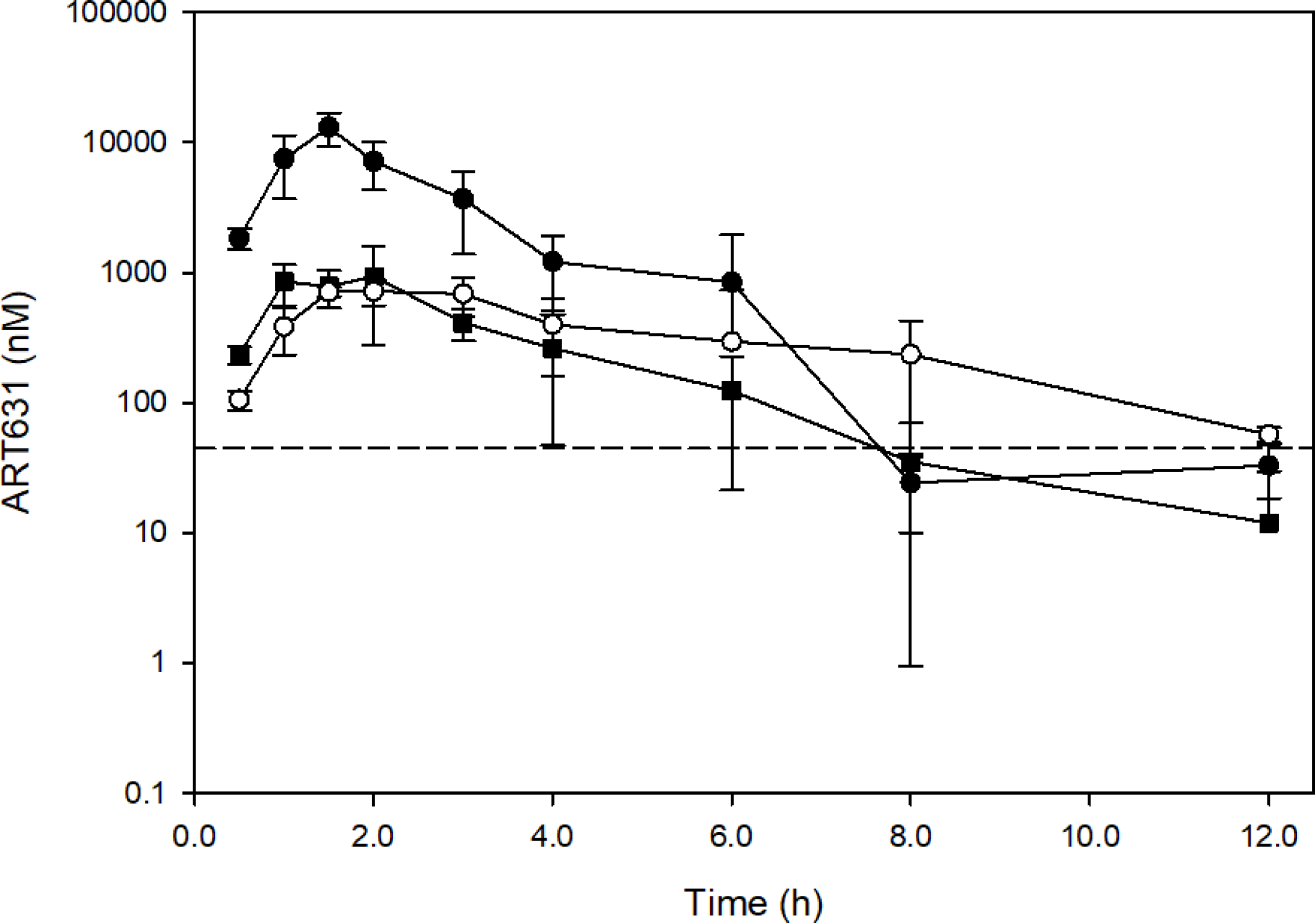
Concentration-time profiles of ART631 in NRG mice (n=3) treated with single doses of 75 mg/kg PO ART631 (MTD dose; closed circle), or multiple doses of 15 mg/kg PO ART631 for 5 days (MTD dose) alone (open circle) or in the SAV combination (closed square). Plasma was obtained over 12h, with ART631 concentration determined by LC/MS-MS. Dashed line represents the highest *in vitro* IC_50_ of ART631 (45 nM). Data points and error bars represent mean and SD of 3 replicates, respectively.

Plasma concentration-time profiles following ART631 administration at the single and multiple dose MTD and in the SAV combination are presented in **Figure 6**, and associated PK parameters in **Table 4**. After PO administration, C_max_ occurred at 1.5-2.0h with a mono-exponential decline. The concentration of ART631 exceeded the *in vitro* IC_50_ for 9 of the 10 AML cell lines tested (45 nM) for at least 6h after administration as a single dose, as multiple doses alone, or as multiple doses in the SAV combination (**Figure 6**). ART631’s half-life of 1.1 - 1.7h (alone) - 2.4h (in SAV) indicates that daily dosing is reasonable. Using the Method of Bailer, the AUC_last_ from single dose ART631 (22905 nM*h) was significantly higher than AUC_last_ from multiple doses of ART631, either alone (2737 nM*h) or when administered as part of the SAV combination (3734 nM*h) (**Table 4**). The AUC_last_ from multiple dose ART631 when administered in SAV (3734 nM*h) was significantly higher compared to that of the multiple dose ART631 administered alone (2737 nM*h) (**Table 4**), but this increased exposure did not result in increased clinical toxicity.

**Table 4:** PK parameters for 2C-ARTs after single dose and five-day oral regimens.

Drug	Dose	Schedule	C_max_	T_max_	AUC_last_	T_1/2_	Vz/F	CL/F
	(mg/kg/d)		(nM)	(h)	(nM*h)	(h)	(L/kg)	(L/h/kg)
**ART631**	75	PO x 1	13063	1.5	22905	1.1	8.4	5.2
**ART631**	15	PO qd x 5	935	2.0	2737	1.7	21.5	8.7
**ART631 in SAV**	15	PO qd x 5	719	2.0	3734	2.4	21.3	6.1

Pharmacokinetics were assessed in the blood plasma of NRG mice after administration of each of the noted dosing regimens (75 mg/kg ART631 PO x 1, 15 mg/kg ART631 PO qd x 5d both alone and in SAV). For reference, the maximal tolerated dose (MTD) of ART631 was determined to be 75 mg/kg PO x 1 or 15 mg/kg/d PO x 5d. Plasma was collected at multiple time points 0.5-12 hours after the single regimens or after the 5^th^ dose of the five-day regimens. Samples were analyzed by liquid chromatography/triple quadrupole mass spectrometry (LC/MS-MS) as previously described (17). PK parameters (maximum plasma concentration [C_max_]; time to reach C_max_ [T_max_]; area under the plasma concentration-time curve [AUC] from time zero to the last measurable concentration [AUC_last_]; plasma half-life [T_1/2_]; apparent volume of distribution [Vz/F]; apparent clearance [CL/F]) were calculated from mean drug concentration-time data using non-compartmental methods as analyzed in Phoenix WinNonlin version 8.3.

### ART631-Containing SAV Induced Deep, Long Remissions in Two Human AML Cell Line Xenograft Models

**Figure 7:**
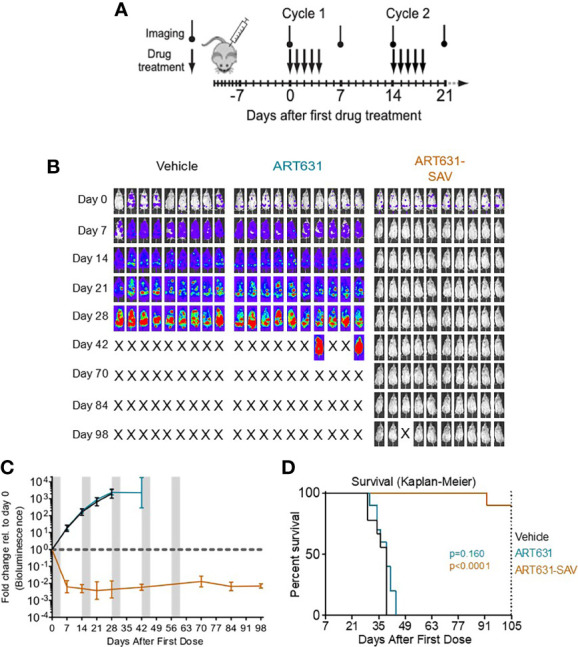
VEN plus SOR plus ART631 induced deep, long remissions in AML xenografts. **(A)** SAV MTD cyclic drug treatment schema. **(B)** NRG mice were transplanted IV with Luc-labeled MV4;11 cells on day -10. After Xenogen quantification of baseline total body luminescence on day 0, mice were placed into balanced experimental groups prior to drug administration *via* gavage, then treated per our previously established MTD PO x 5 day schedule (5 days on, 9 days off). Mice were treated with 15 mg/kg/d ART631 as monotherapy or in combination with 150 mg/kg/d VEN and 30 mg/kg/d SOR for 5 identical 5-day treatment cycles over 10 weeks. These xenograft experiments with ART631 and ART631-containing SAV were performed simultaneously with those with ART838 and ART838-containing SAV reported previously (20). Treatment response outcomes were measured by **(C)** leukemia response quantitation (fold-change in leukemia burden on day 7 (and later) vs day 0 for each mouse *via* Xenogen imaging (grey-shaded bands on graphs indicate days when mice were treated). Error bars represent SD of fold-change in leukemia burden of mice in each treatment group. **(D)** shows corresponding Kaplan-Meier survival curves. Mouse deaths empirically defined to have occurred for reasons other than leukemia (i.e. deaths of mice with luminescence at or below average background luminescence (5.7x10^5^ photons/mouse based on intensity values from non-leukemia bearing mice) were censored. Comparisons between treatment groups were calculated by log-rank (Mantel-Cox) test.

**Figure 8:**
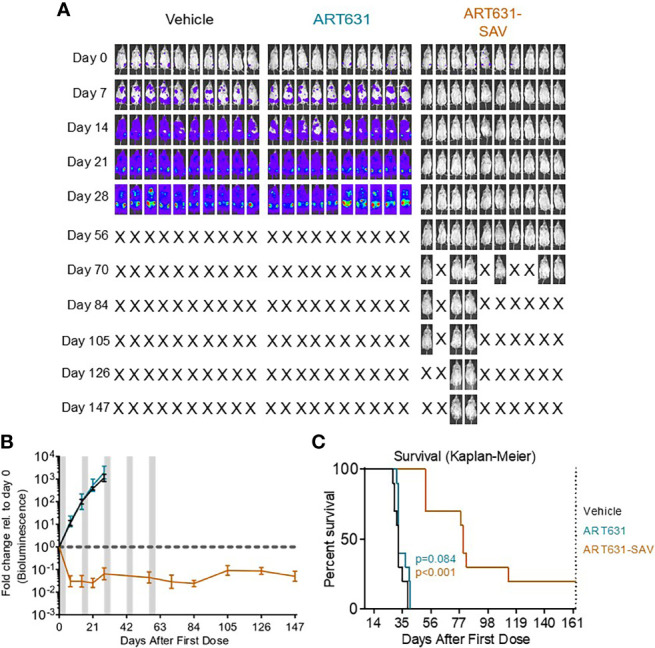
VEN plus SOR plus ART631 induced deep, long remissions in AML xenografts. **(A)** NRG mice were transplanted IV with Luc-labeled MOLM14 cells on day -10. After Xenogen quantification of baseline total body luminescence on day 0, mice were placed into balanced experimental groups prior to drug administration *via* gavage, then treated per our previously established MTD PO x 5 day schedule (5 days on, 9 days off). Mice were treated with 15 mg/kg/d ART631 as monotherapy or in combination with 150 mg/kg/d VEN and 30 mg/kg/d SOR for 5 identical 5-day treatment cycles over 10 weeks. These xenograft experiments with ART631 and ART631-containing SAV were performed simultaneously with those with ART838 and ART838-containing SAV reported previously (20). Treatment response outcomes were measured by **(B)** leukemia response quantitation (fold-change in leukemia burden on day 7 (and later) vs day 0 for each mouse *via* Xenogen imaging (grey-shaded bands on graphs indicate days when mice were treated). Error bars represent SD of fold-change in leukemia burden of mice in each treatment group. **(C)** shows corresponding Kaplan-Meier survival curves. Mouse deaths empirically defined to have occurred for reasons other than leukemia (i.e. deaths of mice with luminescence at or below average background luminescence (5.7x10^5^ photons/mouse based on intensity values from non-leukemia bearing mice) were censored. Comparisons between treatment groups were calculated by log-rank (Mantel-Cox) test.

Using our previously determined 5-day MTD SAV drug schedule (i.e. 5-day treatment cycles consisting of 15 mg/kg/d ART631 combined with 150 mg/kg/d VEN and 30 mg/kg/d SOR, followed by 9 days off drug; schematic in **Figure 7A**), we assessed treatment of established human luc-labeled MV4;11 (**Figures 7B–D**) and MOLM14 AML (**Figures 8A–C**) cell line xenografts with VEN plus SOR plus ART631 in NRG mice (**Figures 7B**, **8A**). These xenograft experiments with ART631 and ART631-containing SAV were performed simultaneously with those with ART838 and ART838-containing SAV reported previously (20). Outcome metrics included *in vivo* antileukemic efficacy after completion of each treatment cycle relative to treatment day 0, measured as fold-change in AML burden *via* Xenogen imaging (**Figures 7C**, **8B**), and survival (**Figures 7D**, **8C**).

Although no activity of ART631 monotherapy was observed in these experiments, ART631 was clinically tolerable, and the ART631-containing SAV regimen had potent antileukemic efficacy, comparable to that seen with the ART838-containing SAV regimen (20). In the MV4;11 xenograft model, 9 of 10 mice from the ART631-containing SAV group had undetectable AML burdens on day 98 (**Figure 7B**), compared with 5 of 9 mice from the ART838-containing SAV group (20); however, the increase in survival in the ART631-containing SAV group compared with the ART838-containing SAV group did not reach statistical significance (p=0.112). In the MOLM14 xenograft model, 2 of 10 mice from the ART631-containing SAV group had undetectable AML burdens on day 147 (**Figure 8A**), compared with 2 of 9 mice from the ART838-containing SAV group (20). Treatment ended on day 60 for both MV4;11 and MOLM14 xenograft models.

### ART631-Containing SAV Slowed Growth of an AML PDX Model

**Figure 9:**
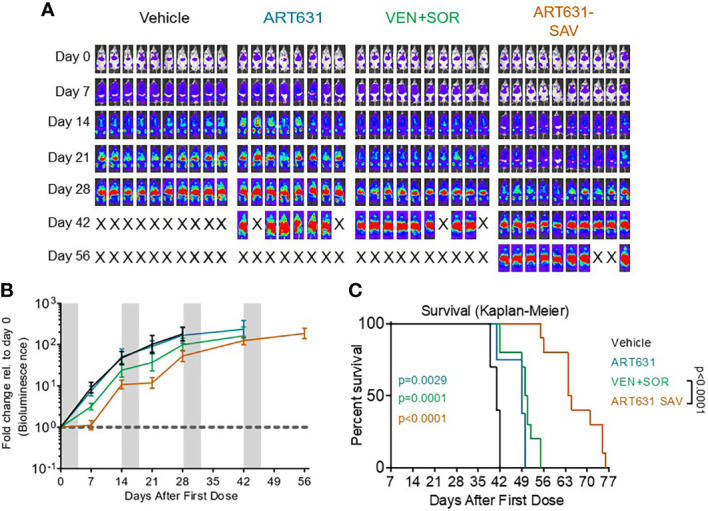
VEN plus SOR plus ART631 inhibited growth of an AML PDX. NRG mice bearing luc/YFP-labeled AML45 PDX were prepared as in **Figure 7**. **(A)** After Xenogen quantification of baseline total body luminescence on day 0, mice were placed into balanced experimental groups prior to drug administration *via* gavage, then treated per our previously established MTD PO x 5 day schedule (5 days on, 9 days off). Mice were treated with 15 mg/kg/d ART631 as monotherapy, with 150 mg/kg/d VEN and 30 mg/kg/d SOR, or with ART631 plus VEN plus SOR (i.e. SAV) at the previously indicated doses for 4 identical 5-day treatment cycles over 8 weeks. This PDX experiment was also performed simultaneously with the previously reported AML45 PDX experiment with ART838 and ART838-containing SAV (20). Treatment response outcomes were measured by **(B)** leukemia response quantitation (fold-change in leukemia burden on day 7 (and later) versus day 0 for each mouse *via* Xenogen imaging (grey-shaded bands on graphs indicate days when mice were treated). Error bars represent the SD of fold-change in leukemia burden of mice in each treatment group. **(C)** shows corresponding Kaplan-Meier survival curves. Comparisons between treatment groups were calculated by log-rank (Mantel-Cox) test.

We then compared the efficacy of 15 mg/kg/d ART631 monotherapy, versus 150 mg/kg/d VEN plus 30 mg/kg/d SOR, versus ART631 combined with VEN plus SOR (i.e. SAV) at the previously indicated doses, using a luc-labeled AML45 PDX model (**Figure 9A**). This PDX experiment was performed simultaneously with the previously reported AML45 PDX experiment evaluating ART838 monotherapy and ART838-containing SAV (20). All combination regimens [SOR plus VEN, ART631-containing SAV, and ART838-containing SAV (20)] slowed the increase in this patient-derived AML burden over time versus vehicle (**Figure 9B**). ART631 monotherapy resulted in a statistically significant increase in survival versus vehicle (p=0.0029; **Figure 9C**), though the magnitude was small; whereas the increase in survival did not reach statistical significance with ART838 monotherapy versus vehicle (20). Both SAV groups had longer survival than SOR plus VEN (p=0.0002 for ART838-containing SAV (20) and p<0.0001 for ART631-containing SAV; **Figure 9C**). The ART631-containing SAV group had longer survival than the ART838-containing SAV group (p=0.0187) and a nearly twofold survival increment over vehicle (**Figure 9C**).

## Discussion

ART838 was previously shown to have superior potency against AML *in vitro* and *in vivo*, as compared to AS, as monotherapy (17). ART838 was also shown to synergize with both VEN and SOR *in vitro* and *in vivo* (17, 20), most notably in xenograft models (20), and to have superior pharmacologic parameters (17). However, ART838 is nearing the end of its patent life, reducing its appeal for the commercial investment necessary to develop a new drug.

The class of 2C-ARTs offered promise, as they were predicted to have similar or better pharmacologic properties compared to ART838, based on their lower molecular weights, improved functionality, and adherence to Lipinski’s rules. Indeed, multiple 2C-ART derivatives had previously demonstrated potent *in vivo* efficacy in a malaria model (19), suggesting good drug-like pharmacology. Their extended patent protection, physicochemical features, and *in vivo* antimalarial activity indicated that 2C-ART derivatives might be more attractive for development as antineoplastics, compared to earlier ART derivatives including ART838. We therefore wished to identify a new, commercially viable 2C-ART with potential to be a key component of a novel, effective, low-toxicity AML treatment paradigm and a valuable addition to the current limited antileukemic therapeutic armamentarium. We selected two 2C-ARTs — ART576 and ART631— for evaluation in comparison to ART838, because of their *in vivo* antimalarial activity and ease of synthesis.

ART631 was more active than ART576 and had similar *in vitro* activity as ART838 against multiple AML cell lines (**Table 2**). Synergism of ART838 with SOR and VEN was formally reported *in vitro* previously (20). Here, we set out evaluate whether ART631 adds to SOR and VEN, and our results indicate that these 3 drugs cooperate to enhance apoptotic AML cell death (**Figure 3**). The amount of drug cooperativity of the ART631-containing SAV 3-drug combination may have been obscured in these assays by the high activity of the 2-drug SOR plus VEN and ART631 plus VEN combinations (20). ART631-containing SAV was potently active in the two tested AML cell line xenograft models, causing deep responses in MV4;11 and MOLM14 xenografts at day 7, which persisted for months during and after treatment completion and were overall comparable to responses seen in simultaneous experiments with ART838-containing SAV in xenograft models (20). Strikingly, SAV with ART631 appears to have cured 9 of 10 MV4;11 xenografted mice (**Figure 7**) and 2 of 10 MOLM14 xenografted mice (**Figure 8**). ART631-containing SAV was also similarly clinically tolerable compared with ART838-containing SAV (20). It is important to note here that we demonstrated previously that VEN plus SOR was extremely potent against MV4;11 and MOLM14 xenografts, possibly reducing the demonstrable additive or synergistic contributions of both ART631 and ART838 in their respective SAV regimens (20).

In contrast to the impressive sensitivity of these two AML cell line xenograft models to SAV regimens containing either ART631 or ART838, the AML45 PDX model, which was derived from an older adult with relapsed, refractory AML (20), was much less sensitive to all tested drug combinations. However, ART631-containing SAV was clearly the most leukemia growth inhibitory regimen and resulted in longer survival than both ART838-containing SAV (20) and the SOR plus VEN 2-drug combination (**Figure 9C**). These *in vivo* results in the AML PDX model confirm the observed *in vitro* cooperativity between the SAV drugs as well as the contribution of ART631 to the SAV regimen in a subtype of AML that is notoriously resistant to treatment. We propose that SAV containing ART631 (or potentially other 2C-ARTs) offers a good starting point for a future novel, low-toxicity treatment paradigm for AML that can potentially be improved upon with either additional synergistic agents or with substitutions for either SOR or VEN; namely, with more potent and selective kinase and BCL2 inhibitors, respectively (26–29).

ART631 was acceptably stable in microsomes, though it lacked the robust stability of ART838 (17). ART631 exhibited similar pharmacokinetics as ART838; the multiple day MTD regimen of ART631 (15 mg/kg/d x5) resulted in similar exposure (**Figure 6** and **Table 4**) as that previously shown for the multiple day MTD regimen of ART838 (50 mg/kg/d x5) (20). When administered as part of SAV, ART631 exposure was somewhat higher and half-life longer compared with ART631 monotherapy (**Figure 6** and **Table 4**), though fortunately increased toxicity was not observed.

While it is somewhat limited in its *in vitro* stability, ART631 is a first-in-class antileukemic 2C-ART with potent activity *in vitro* and *in vivo*, is easily synthesized, and is highly effective, at least serving here as a “tool” compound for proof-of-principle experiments demonstrating the efficacy of a 2C-ART as a component of SAV. Lead optimization studies can now be performed to improve upon the efficacy, stability, and pharmacokinetic properties of ART631 or potentially other 2C-ARTs, in an effort to identify a lead 2C-ART. This new lead 2C-ART will be an essential component of a novel combination regimen that warrants clinical testing in leukemia patients.

## Data Availability

The raw data supporting the conclusions of this article will be made available by the authors, without undue reservation.

## References

[1] Kantarjian HM Short NJ Fathi AT Marcucci G Ravandi F Tallman M. Acute Myeloid Leukemia: Historical Perspective and Progress in Research and Therapy Over 5 Decades. Clin Lymphoma Myeloma Leuk (2021) 21(9):580–97. doi: 10.1016/j.clml.2021.05.016 PMC11938811 34176779

[2] Sasaki K Ravandi F Kadia TM DiNardo CD Short NJ Borthakur G. *De Novo* Acute Myeloid Leukemia: A Population-Based Study of Outcome in the United States Based on the Surveillance, Epidemiology, and End Results (SEER) Database 1980 to 2017. Cancer (2021) 127(12):2049–61. doi: 10.1002/cncr.33458 PMC11826308 33818756

[3] Lai C Doucette K Norsworthy K. Recent Drug Approvals for Acute Myeloid Leukemia. J Hematol Oncol (2019) 12(1):100. doi: 10.1186/s13045-019-0774-x 31533852 PMC6749668

[4] Konopleva M Pollyea DA Potluri J Chyla B Hogdal L Busman T. Efficacy and Biological Correlates of Response in a Phase II Study of Venetoclax Monotherapy in Patients With Acute Myelogenous Leukemia. Cancer Discov (2016) 6(10):1106–17. doi: 10.1158/2159-8290.CD-16-0313 PMC5436271 27520294

[5] Scholl S Fleischmann M Schnetzke U Heidel FH. Molecular Mechanisms of Resistance to FLT3 Inhibitors in Acute Myeloid Leukemia: Ongoing Challenges and Future Treatments. Cells (2020) 9(11):2493. doi: 10.3390/cells9112493 PMC7697863 33212779

[6] McMurry H Fletcher L Traer E. IDH Inhibitors in AML-Promise and Pitfalls. Curr Hematol Malig Rep (2021) 16(2):207–17. doi: 10.1007/s11899-021-00619-3 33939107

[7] Rudrapal M Chetia D. Endoperoxide Antimalarials: Development, Structural Diversity and Pharmacodynamic Aspects With Reference to 1,2,4-Trioxane-Based Structural Scaffold. Drug Des Devel Ther (2016) 10:3575–90. doi: 10.2147/DDDT.S118116 PMC5098533 27843298

[8] Keiser J Utzinger J. Artemisinins and Synthetic Trioxolanes in the Treatment of Helminth Infections. Curr Opin Infect Dis (2007) 20(6):605–12. doi: 10.1097/QCO.0b013e3282f19ec4 17975411

[9] Arav-Boger R He R Chiou CJ Liu J Woodard L Rosenthal A. Artemisinin-Derived Dimers Have Greatly Improved Anti-Cytomegalovirus Activity Compared to Artemisinin Monomers. PloS One (2010) 5(4):e10370. doi: 10.1371/journal.pone.0010370 20442781 PMC2860993

[10] He R Mott BT Rosenthal AS Genna DT Posner GH Arav-Boger R. An Artemisinin-Derived Dimer has Highly Potent Anti-Cytomegalovirus (CMV) and Anti-Cancer Activities. PloS One (2011) 6(8):e24334. doi: 10.1371/journal.pone.0024334 21904628 PMC3164168

[11] Efferth T Dunstan H Sauerbrey A Miyachi H Chitambar CR. The Anti-Malarial Artesunate Is Also Active Against Cancer. Int J Oncol (2001) 18(4):767–73. doi: 10.3892/ijo.18.4.767 11251172

[12] Efferth T Sauerbrey A Olbrich A Gebhart E Rauch P Weber HO. Molecular Modes of Action of Artesunate in Tumor Cell Lines. Mol Pharmacol (2003) 64(2):382–94. doi: 10.1124/mol.64.2.382 12869643

[13] Hooft van Huijsduijnen R Guy RK Chibale K Haynes RK Peitz I Kelter G. Anticancer Properties of Distinct Antimalarial Drug Classes. PloS One (2013) 8(12):e82962. doi: 10.1371/journal.pone.0082962 24391728 PMC3877007

[14] Choo EF Boggs J Zhu C Lubach JW Catron ND Jenkins G. The Role of Lymphatic Transport on the Systemic Bioavailability of the Bcl-2 Protein Family Inhibitors Navitoclax (ABT-263) and ABT-199. Drug Metab Dispos (2014) 42(2):207–12. doi: 10.1124/dmd.113.055053 24212376

[15] Efferth T Verdorfer I Miyachi H Sauerbrey A Drexler HG Chitambar CR. Genomic Imbalances in Drug-Resistant T-Cell Acute Lymphoblastic CEM Leukemia Cell Lines. Blood Cells Mol Dis (2002) 29(1):1–13. doi: 10.1006/bcmd.2002.0530 12482398

[16] Drenberg CD Buaboonnam J Orwick SJ Hu S Li L Fan Y. Evaluation of Artemisinins for the Treatment of Acute Myeloid Leukemia. Cancer Chemother Pharmacol (2016) 77(6):1231–43. doi: 10.1007/s00280-016-3038-2 PMC4918815 27125973

[17] Fox JM Moynihan JR Mott BT Mazzone JR Anders NM Brown PA. Artemisinin-Derived Dimer ART-838 Potently Inhibited Human Acute Leukemias, Persisted In Vivo, and Synergized With Antileukemic Drugs. Oncotarget (2016) 7(6):7268–79. doi: 10.18632/oncotarget.6896 PMC4872784 26771236

[18] Budhraja A Turnis ME Churchman ML Kothari A Yang X Xu H. Modulation of Navitoclax Sensitivity by Dihydroartemisinin-Mediated MCL-1 Repression in BCR-ABL(+) B-Lineage Acute Lymphoblastic Leukemia. Clin Cancer Res (2017) 23(24):7558–68. doi: 10.1158/1078-0432.CCR-17-1231 PMC5786379 28974549

[19] Mott BT Tripathi A Siegler MA Moore CD Sullivan DJ Posner GH. Synthesis and Antimalarial Efficacy of Two-Carbon-Linked, Artemisinin-Derived Trioxane Dimers in Combination With Known Antimalarial Drugs. J Med Chem (2013) 56(6):2630–41. doi: 10.1021/jm400058j PMC3630999 23425037

[20] Moses BS McCullough S Fox JM Mott BT Bentzen SM Kim M. Antileukemic Efficacy of a Potent Artemisinin Combined With Sorafenib and Venetoclax. Blood Adv (2021) 5(3):711–24. doi: 10.1182/bloodadvances.2020003429 PMC7876886 33560385

[21] Zimmerman EI Turner DC Buaboonnam J Hu S Orwick S Roberts MS. Crenolanib Is Active Against Models of Drug-Resistant FLT3-ITD-Positive Acute Myeloid Leukemia. Blood (2013) 122(22):3607–15. doi: 10.1182/blood-2013-07-513044 PMC3837509 24046014

[22] Rudek MA Zhao M Smith NF Robey RW He P Hallur G. *In Vitro* and In Vivo Clinical Pharmacology of Dimethyl Benzoylphenylurea, A Novel Oral Tubulin-Interactive Agent. Clin Cancer Res (2005) 11(23):8503–11. doi: 10.1158/1078-0432.CCR-05-1037 16322314

[23] Bailer AJ. Testing for the Equality of Area Under the Curves When Using Destructive Measurement Techniques. J Pharmacokinet Biopharm (1988) 16(3):303–9. doi: 10.1007/BF01062139 3221328

[24] Yuan J. Estimation of Variance for AUC in Animal Studies. J Pharm Sci (1993) 82(7):761–3. doi: 10.1002/jps.2600820718 8360854

[25] Efferth T Giaisi M Merling A Krammer PH Li-Weber M. Artesunate Induces ROS-Mediated Apoptosis in Doxorubicin-Resistant T Leukemia Cells. PloS One (2007) 2(8):e693. doi: 10.1371/journal.pone.0000693 17668070 PMC1933253

[26] Pan R Ruvolo VR Wei J Konopleva M Reed JC Pellecchia M. Inhibition of Mcl-1 With the Pan-Bcl-2 Family Inhibitor (-)BI97D6 Overcomes ABT-737 Resistance in Acute Myeloid Leukemia. Blood (2015) 126(3):363–72. doi: 10.1182/blood-2014-10-604975 PMC4504949 26045609

[27] Khaw SL Suryani S Evans K Richmond J Robbins A Kurmasheva RT. Venetoclax Responses of Pediatric ALL Xenografts Reveal Sensitivity of MLL-Rearranged Leukemia. Blood (2016) 128(10):1382–95. doi: 10.1182/blood-2016-03-707414 PMC5016707 27343252

[28] Nguyen B Williams AB Young DJ Ma H Li L Levis M. FLT3 Activating Mutations Display Differential Sensitivity to Multiple Tyrosine Kinase Inhibitors. Oncotarget (2017) 8(7):10931–44. doi: 10.18632/oncotarget.14539 PMC5355235 28077790

[29] Tarver TC Hill JE Rahmat L Perl AE Bahceci E Mori K. Gilteritinib Is a Clinically Active FLT3 Inhibitor With Broad Activity Against FLT3 Kinase Domain Mutations. Blood Adv (2020) 4(3):514–24. doi: 10.1182/bloodadvances.2019000919 PMC7013266 32040554

